# Hybrid machine learning framework for electrical grid stability prediction using comparative model evaluation

**DOI:** 10.3389/frai.2026.1963509

**Published:** 2026-09-23

**Authors:** N. Subramanian, Albert Alexander Stonier

**Affiliations:** School of Electrical Engineering, Vellore Institute of Technology, Vellore, India

**Keywords:** deep neural networks, explainable AI, hybrid machine learning, smart grid stability, stacking ensemble, XGBoost

## Abstract

**Introduction:**

Accurately predicting electrical grid stability is essential to ensuring the reliability, resilience, and sustainable operation of modern smart energy systems. The nonlinear interactions among the grid operating parameters make stability prediction challenging, particularly when conventional machine learning models depend primarily on statistical relationships, without clearly incorporating physically meaningful characteristics.

**Methods:**

This study presents a systematic comparative evaluation of machine-learning and deep-learning approaches for electrical grid stability prediction using the Electrical Grid Stability Simulated Dataset, comprising 10,000 observations from a decentralized four-node star-topology power system. Six conventional machine learning models - Logistic Regression, Random Forest, Gradient Boosting, XGBoost, LightGBM, and Support Vector Machine with an RBF kernel are compared with four deep-learning architectures, namely Deep Multilayer Perceptron, 1D-CNN, CNN-LSTM, and Bi-LSTM. We also develop a hybrid stacking ensemble to integrate complementary representations from high-performing boosting models through a linear meta-learner. To incorporate domain knowledge, seven physics-informed features are engineered from the original 12 grid parameters, producing a 19-dimensional feature space. Model performance is evaluated using accuracy, weighted F1-score, ROC- AUC, Matthews correlation coefficient, and class-wise precision and recall, evaluated using stratified fivefold cross-validation. SHAP-based analysis is employed to quantify feature contributions and improve model interpretability.

**Results:**

The proposed hybrid stacking ensemble achieves the best overall predictive performance, obtaining an accuracy of 96.85%, weighted F1 score of 0.9685, ROC-AUC of 0.9953, and MCC of 0.9319. The results demonstrate that integrating complementary ensemble learners provides more reliable stability discrimination than individual conventional and deep learning models. SHAP analysis further indicates that physics- informed interaction features, particularly τ−*P* interaction and power balance characteristics, provide substantial discriminatory information when combined with the original grid reaction time parameters.

**Discussion:**

The findings demonstrate that hybrid ensemble learning combined with physics-informed feature engineering will provide accurate, interpretable, and computationally practical solutions for electrical grid stability prediction. The strong predictive discrimination and consistent cross-validation performance indicate its potential for real-time stability monitoring and decision support in smart-grid environments. The integration of domain-informed representations with data-driven learning offers a promising direction for developing more transparent and reliable intelligent grid-monitoring systems.

## 1 Introduction

The operational characteristics of modern electrical grids have changed due to the rapid development of renewable energy and the increasing use of decentralized energy resources. Unlike conventional power systems, which are characterized by large-scale synchronous machines, distributed generators, prosumers and price-responsive energy markets present distinctive characteristics. To maintain system stability during disruptions, conventional generators integrate inertia and frequency support. Distributed energy units, on the other hand, typically operate with different control systems and response times. These variations increase the possibility of instability after unexpected changes in load or generation, and this will result in complex interconnections throughout the network. As a result, the most important requirement for safe grid operation is precise and timely grid stability prediction.

Physics modeling of power system dynamics is the main foundation of conventional stability evaluation techniques. Methods like eigenvalue analysis and small signal stability evaluation depend on linearized network representations and they require a deep understanding of system architecture and features. These methods are still important for power system analysis, but they are computationally challenging, and it is not suitable for real-time monitoring in large, highly dynamic systems. The use of data-driven methods for stability prediction has increased due to the growing availability of operational and simulated data. After the model is built, machine learning techniques could very quickly classify data based on patterns linked to both stable and unstable operating conditions. Based on recent studies, nonlinear classifiers and ensemble learning techniques will capture the complex relationships between grid configurations and stability results. A comprehensive comparison of various machine learning algorithms under reliable environmental conditions is still limited. Furthermore, much of current research focuses only on initial grid settings without investigating feature engineering techniques that capture actual user interactions within the network. Research on how artificial characteristics affect model interpretability and prediction performance is still important. This study uses the Electrical Grid Stability Simulated Dataset (Reksoprodjo, 2021), which analyzes the behavior of a decentralized four-node star topology power system, to evaluate multiple machine learning and hybrid ensemble models to address these issues. To capture system-level energy balance and interaction effects, several physics-inspired design elements have been incorporated into the fundamental grid characteristics. Additionally, a Hybrid Stacking Ensemble that combines Random Forest, XGBoost, Light GBM, and Logistic Regression inside the meta learning framework is shown in the article. Finally, SHAP-based interpretability analysis is used to determine the characteristics that significantly affect stability prediction and to determine the relative importance of the input features.

## 2 Literature review

The rapid integration of distributed generation, intelligent communication infrastructures, and renewable energy sources has made modern power systems more complex. Because of these advancements, new operational difficulties have emerged, especially when it comes to preserving system stability and predictable dynamic environments. Conventional stability evaluation techniques mostly depend on analytical models and in-depth time domain simulations, which are computationally demanding and frequently impractical for real-time monitoring. As a result, deep learning (DL) and machine learning (ML) approaches have become effective alternatives for rapid and data-driven stability prediction. According to recent studies (Alimi et al., 2020; Sarajcev et al., 2022), ML-based approaches will analyze large amounts of operational data efficiently and offer faster decision-making than traditional simulation-based methods.Employing deep learning models for transient and voltage stability evaluation has been the focus of a significant amount of study. It has been demonstrated that convolutional neural networks (CNNs) will successfully identify instability modes in power systems and extract spatial patterns from system measurements (Shi et al., 2020). In the same manner, deep neural networks have proven to be highly effective at capturing nonlinear correlations between voltage stability indicators and system variables (Zhang et al., 2021). To better predict complex power systems with high integration of power electronic devices and renewable generation, hybrid architectures that combine convolutional and recurrent neural networks have also been recommended to capture both spatial and temporal dependencies in system dynamics (Tapia et al., 2022; Li et al., 2022). For grid stability prediction, several studies have adopted ensemble methods and conventional machine learning models in addition to deep learning approaches. Using developed features obtained from system measurements, algorithms like Support Vector Machines, and Random Forest and Gradient Boosting have shown competitive performance in identifying stability conditions. By mixing several base learners, ensemble learning techniques have been demonstrated to increase prediction accuracy. According to research on voltage stability monitoring and transient stability prediction, ensemble-based methods will successfully capture complicated nonlinear interactions while retaining dependable performance across various operating conditions (Dharmaapala et al., 2020; Aygul et al., 2024). Improving machine learning models, adaptability and interpretability in power system applications is a significant area of research. By allowing models trained on one network configuration to be converted to another, transfer learning techniques have been proposed to meet the difficulty of changing grid topologies and operational conditions (Hijazi et al., 2023). Simultaneously, automated machine learning methods have been developed to improve model transparency and interpretability while optimizing feature selection hyperparameter tuning. These methods aim to improve the practicality of ML models in operational settings where explainability and dependability are essential (Liu F. et al., 2023). A suitable approach for integrating physical understanding of power system dynamics into data-driven models is physics-informed machine learning (PIML), which has emerged more recently. Differential equations and system restrictions are directly incorporated into the learning process by physics-informed neural networks, which improve model generalization and reduce dependence on large data sets (Huang and Wang, 2022). When compared to data-driven methods, these approaches have shown improved precision and physical uniformity in probabilistic analysis, voltage stability monitoring and state estimation issues (Ye et al., 2022; Lu and Bu, 2024; Su et al., 2023; Ngo et al., 2024; Mohammadian et al., 2023; Nellikath et al., 2024; Huang and Wang, 2022; Liu Y. et al., 2023; Liu et al., 2025; Massoudi et al., 2024). Even though ML and DL based stability evaluation methods have advanced significantly, there are still several issues that need to be addressed, such as restricted interpretability, sensitivity to data quality, and limitations in adjusting models to rapidly fluctuating grid conditions. The reliability of data-driven models has been improved by physics-informed learning and graph-based neural networks, but the combination of hybrid ensemble methods and physics-informed feature engineering is still largely unexplored. Therefore, reliable hybrid machine learning frameworks that will provide accurate, comprehensible and real-time stability prediction continues to be an important component of research for upcoming smart grid applications.

## 3 Dataset description

### 3.1 Physical model

A simplified decentralized power system with a symmetric four-node star topology is represented by the electrical grid stability simulated dataset (Reksoprodjo, 2021). It is a decentralized consumer-producer network made up of one generator node linked to three consumer nodes. A set of coupled second-order differential equations that characterize the systems frequency deviation response to changes in power imbalance and price-driven demand response controls the grid's dynamic behavior. The maximum real section of the eigenvalue spectrum, represented by λ_max_, determines the stability of the system. The operational point is considered stable when λ_max_ < 0 and system perturbations gradually diminish. On the other hand, disturbances increase exponentially if λ_max_>0, indicating an unstable grid state. The dataset labels used in the classification challenge are generated based on this stability requirement.

### 3.2 Feature description

The dataset has 10,000 simulated observations that are characterized by 14 features that correspond to the decentralized grids operational parameters. These characteristics represent price elasticity coefficients, nominal power exchange levels, and the participant reaction times, all of which together govern the systems dynamic behavior. The entire list of attributes, including their physical interpretation, measurement units, and value ranges, is summarized in Table 1. The maximum real eigenvalue derived from the stability study is represented by the variable *stab* as shown in Table 2. To avoid information leaking, the continuous *stab* variable is not included in model training since the categorical label *stabf* is directly generated from the sign of this value.

**Table 1:** Dataset feature description.

Feature	Type	Range	Unit	Physical description
tau1	Real	0.5–10	s	Reaction time of producer node 1
tau2	Real	0.5–10	s	Reaction time of consumer node 2
tau3	Real	0.5–10	s	Reaction time of consumer node 3
tau4	Real	0.5–10	s	Reaction time of consumer node 4
p1	Real	0.5–5.5	p.u.	Nominal power produced by node 1 (+)
p2	Real	–2 to –0.5	p.u.	Nominal power consumed by node 2 (–)
p3	Real	–2 to –0.5	p.u.	Nominal power consumed by node 3 (–)
p4	Real	–2 to –0.5	p.u.	Nominal power consumed by node 4 (–)
g1	Real	0.05–1.0	–	Price elasticity coefficient for node 1
g2	Real	0.05–1.0	–	Price elasticity coefficient for node 2
g3	Real	0.05–1.0	–	Price elasticity coefficient for node 3
g4	Real	0.05–1.0	–	Price elasticity coefficient for node 4
stab	Real	–0.08 to 0.11	s^−1^	Maximum real eigenvalue (excluded from training)
stabf	Categorical	Stable/unstable	–	Binary stability label derived from *stab*

**Table 2:** Sample observations from the electrical grid stability dataset.

ID	tau1	tau2	tau3	tau4	p1	p2	p3	p4	g1	g2	g3	g4	stab	stabf
0	2.95	3.07	8.38	9.78	3.76	–0.78	–1.26	–1.72	0.65	0.86	0.89	0.96	0.06	Unstable
1	9.30	4.90	3.05	1.37	5.07	–1.94	–1.87	–1.26	0.41	0.86	0.56	0.78	–0.01	Stable
2	8.97	8.85	3.05	1.21	3.41	–1.21	–1.28	–0.92	0.16	0.77	0.84	0.11	0.00	Unstable
3	0.71	7.67	4.49	2.34	3.96	–1.03	–1.94	–1.00	0.44	0.98	0.93	0.36	0.03	Unstable
4	3.13	7.61	4.94	9.86	3.53	–1.13	–1.85	–0.55	0.80	0.46	0.66	0.82	0.05	Unstable

### 3.3 Class distribution

There is an average of class imbalance between stable and unstable operational states in the sample. Three thousand six hundred twenty samples (36.2%) of the 10,000 observations relate to stable grid states, whereas 6,380 samples (63.8%) correspond to unstable conditions. As a result, the imbalance ratio is approximately 1.76:1. Accuracy alone cannot be used to accurately evaluate categorization performance given this distribution. Without identifying any stability patterns, a basic classifier that classifies all samples as unstable would appear to have an accuracy of 63.8%. Because it offers a fair evolution of classifier performance under unbalanced class distributions, the Matthew's correlation coefficient (MCC) is chosen as the main evaluation metric.

## 4 Methodology

### 4.1 Feature engineering

To capture system-level behavior not clearly reflected by individual node variables, seven design features are added to the initial twelve grid parameters. These characteristics have been designed to represent overall network characteristics, including market sensitivity, response heterogeneity and power balance. The designed features give the learning algorithms more descriptive information by combining interactions between physical variables. The definitions and interpretations of the derived characteristics will be found in Table 3. Mainly, the interaction feature *tau_p_interaction* combines power imbalance with network response delay, which will intensify instability effects. Likewise, the correlation between market responsiveness and reaction delay is represented by the ratio feature *g_tau_ratio*. Although the raw numerical stability margin was excluded to avoid target leakage, feature engineering still introduces indirect dependencies among predictors. Engineered variables such as the power balance and the interaction between τ and power are derived from fundamental system parameters that also remain in the feature space. Accordingly, these variables will be the alternative feature representations rather than independent physical information, and the resulting residual dependence should be regarded as a limitation of the present feature-engineering strategy.

**Table 3:** Engineered feature descriptions.

Feature	Formula	Physical interpretation	Expected influence
power_balance	∑(*p*_1_+*p*_2_+*p*_3_+*p*_4_)	Net energy imbalance in network	Higher imbalance increases instability risk
tau_mean	Mean(τ_1_, τ_2_, τ_3_, τ_4_)	Average network response time	Larger delay tends to reduce stability
tau_std	Std(τ_1_, τ_2_, τ_3_, τ_4_)	Variation in participant response times	Greater heterogeneity may increase instability
g_mean	Mean(*g*_1_, *g*_2_, *g*_3_, *g*_4_)	Average price elasticity across nodes	Higher elasticity can improve stability
g_std	Std(*g*_1_, *g*_2_, *g*_3_, *g*_4_)	Diversity of price responsiveness	Influence depends on interaction with other parameters
tau_p_interaction	τ_mean_×|*power*_*balance*|	Combined effect of response delay and imbalance	Large values indicate increased instability likelihood
g_tau_ratio	*g*_mean_/(τ_mean_+ϵ)	Market sensitivity relative to response delay	Larger ratio associated with improved stability

### 4.2 Data splitting and scaling

To maintain the original class distribution, stratified random sampling is used to split the dataset into training and testing divisions. Twenty percent of the data (2,000 samples) is designated for evaluation, and the remaining 80 percent (8,000 samples) is used for model training. In both subgroups, this process preserves the ratio of 63.8:36.2 of unstable to stable samples. A common normalization process is used for feature scaling. Only the training data is used to fit a standard scaler, which is then applied to the testing and training sets. This method prevents information from leaking into the scaling parameters from the test partition. The feature matrix is transformed from a two-dimensional structure in the form (*N*,19,1), where each feature is handled as a distinct channel, for deep learning systems that need three-dimensional inputs.

### 4.3 Model architectures

This study evaluates seven model families. A baseline linear classifier with L2 regularization is logistic regression. Three hundred decision trees, each built with a random subset of features at each split, are used in the random forest bagging technique. Two hundred trees with depth-limited learners are used in the traditional Gradient Boosting approach. To control model complexity, XGBoost includes regularization factors and uses gradient boosting with second-order optimization of the logistic loss function. LightGBM improves computing efficiency for structured data by integrating a histogram-based learning method with leaf-wise tree development. To support nonlinear decision boundaries in high-dimensional feature space, support vector machines with a radial basis function kernel are also evaluated. Finally, a hybrid stacking ensemble combines many base learners. Level 0 models in this architecture include Random Forest, XGBoost, LightGBM and Logistic Regression. A logistic regression meta learner trained on out-of-fold predictions produced by cross-validation is used to combine its predictions.

Hyperparameter tuning was performed using grid-search optimization as shown in Table 4. To avoid data leakage, the optimization was performed within the cross-validation framework. For each cross-validation iteration, hyperparameters were optimized using only the training subset. The optimized model was then evaluated on the held-out fold. As a result, information from the validation folds did not influence hyperparameter selection or model fitting.

**Table 4:** Model hyperparameter summary.

Model	Key hyperparameters	Regularization
Logistic regression	*C* = 1.0, max_iter = 500, solver = lbfgs	L2 penalty
Random Forest	Trees = 300, max_depth = 20, min_samples_leaf = 2	Feature subsampling ($\sqrt{p}$)
Gradient Boosting	Trees = 200, learning_rate = 0.05, depth = 6	Tree depth control
XGBoost	Trees = 400, lr = 0.05, depth = 7, subsample = 0.8	L1 = 0.1, L2 = 1.0
LightGBM	Trees = 400, lr = 0.05, leaves = 63, depth = 10	Column sampling, leaf constraints
SVM (RBF)	*C* = 10, kernel = RBF, gamma = scale	Soft-margin regularization
Hybrid stacking	Base: RF + XGB + LGB + LR; meta: LR (*C* = 5)	Cross-validated meta-learning

### 4.4 Evaluation metrics

On the held-out test set, several complementary parameters are used to assess model performance. A general indicator of categorization reliability is accuracy. The weighted F1 score takes class support into account while striking a balance between recall and precision. Class separation is determined by using the area under the ROC curve (AUC-ROC), which is independent of decision thresholds. The Matthews correlation coefficient (MCC) is the main performance metric because of the data sets moderate class imbalance. Even when class frequencies fluctuate, MCC offers a balanced assessment by using all four components of the confusion matrix. Furthermore, the stable and unstable classes have different reports for precision and recall. Class-specific performance metrics are especially important since interpreting an unstable grid state as stable could increase system risk from an operational standpoint.

## 5 Exploratory data analysis

Before model training, feature distributions and correlations are examined using exploratory analysis. The most important results from several visualization panels are summarized in Figure 1. With roughly 63.8% unstable and 36.2 % stable samples, the class distribution indicates a moderate imbalance. The dataset generation procedure is shown in the almost uniform distributions of the response time variables over the simulated interval of 0.5–10 s. Reaction time (τ), power (*p*), and elasticity (*g*) feature groups show little cross-group relationship, according to correlation analysis. Numerous cross-group correlation coefficients stay below |*r*| < 0.1, indicating a mostly independent sampling of the simulated parameters. The necessity for total generation and consumption will be balanced, leading to negative correlations within the power variables. Near the decision boundary, stable and unstable regions partially overlap in the distribution of the continuous stability variable *stab*. The inability to attain accurate categorization with the available parameters is explained by this overlap. Significant variations in elasticity and power parameters between stability classes are shown by additional visualizations. The application of nonlinear models like kernel techniques and ensemble learners is validated by the interaction plots, which further highlight nonlinear interactions between specific variables.

**Figure 1:**
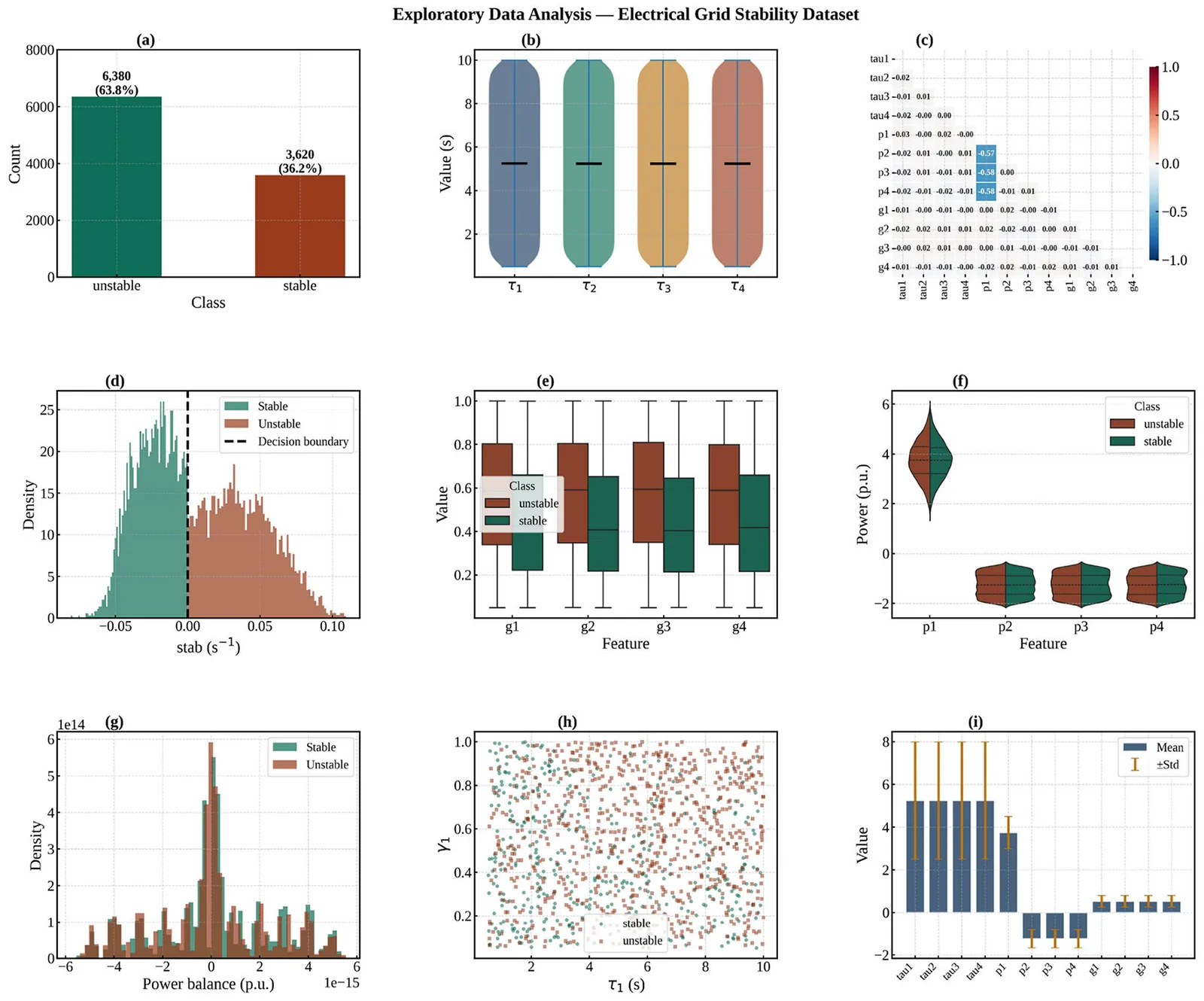
Exploratory data analysis overview showing the distribution and relationships among the grid stability dataset features. **(a)** Class distribution. **(b)** Reaction time distributions. **(c)** Feature correlation matrix. **(d)** Stability value distribution. **(e)** Price elasticity by class. **(f)** Nominal power by class. **(g)** Power balance distribution. **(h)** τ_1_ vs. γ_1_ by class. **(i)** Feature mean ± std.

## 6 Experimental results

### 6.1 Results and discussion

The predictive performance of all examined models on the held-out test set is summarized in Table 5. Figure 2 compares the ROC and classification performance of all models. Nonlinear learning methods provide good classification performance, demonstrating that grid stability patterns will be successfully recorded with the available parameters and constructed features. With an accuracy of 96.85% and a Matthews Correlation Coefficient (MCC) of 0.9319, the Hybrid Stacking Ensemble performs better than all other techniques, demonstrating remarkable agreement between projected and actual class labels despite a small class imbalance. Additionally, the ensemble shows that integrating heterogeneous base learners improves resilience across various regions of the feature space by maintaining balanced precision and recall across both stable and unstable classes. These results suggest that a nonlinear relationship within the constructed tabular feature space will be efficiently learned by fully connected neural networks. With an accuracy of 96.35% and an AUC-ROC of 0.9946 as shown in Figure 3, the SVM with an RBF kernel also exhibits strong performance, demonstrating the capacity of kernel-based techniques to capture complex nonlinear decision limits close to the stability transition region. With the AUC-ROC values greater than 0.99 and accuracy of 95.20% and 95.15%, respectively. XGBoost and LightGBM show almost equal performance profiles among gradient boosting techniques. These models offer an effective choice between computational efficiency and predictive performance. Lower accuracies of 92.30% and 94.60% for classical ensemble techniques like Random Forest and Gradient Boosting indicate that more advanced boosting implementations better capture the nonlinear interactions found in the dataset. Sequential input deep learning architectures perform poorly. While the Bi-LSTM model performs significantly worse with just 81.05% accuracy and an MCC near 0.62, the 1D-CNN and CNN-LSTM models obtain accuracy around 0.90 with MCC values below 0.80. This decrease in performance is predicted as the dataset is made up of single tabular observations rather than temporal sequences, which limits the advantage that recurrent architectures normally deliver. With an accuracy of 87.65% and an MCC of 0.73, logistic regression performs the worst, indicating that the stability boundary in the original feature space is not linearly separable. Further understanding of model behavior is provided by confusion matrix analysis (Figure 4). Higher misclassification rates are shown in linear models, especially when it approaches to their inability to identify unstable states. Nonlinear and ensemble classifiers, on the other hand, significantly lower these significant errors. Only 32 unstable states are incorrectly named as stable by the Deep MLP, which correctly detects 692 stable and 1,244 unstable samples. With 694 stable and 1,243 unstable samples accurately identified and only 33 unstable states wrongly predicted as stable, the Hybrid Stacking Ensemble provides comparable strong results. Figure 5 shows the training and validation curves involved in this models. Moreover, SVM (RBF) achieves balanced classification, properly identifying 690 stable and 1,237 unstable samples while restricting unstable misclassifications to 39 instances. Table 6 shows the proposed model accuracy with previous works. Stability prediction is dominated by designed system-level properties, according to feature important analysis utilizing SHAP (Figures 6, 7). Especially *tau_mean* acts as the most significant predictor, suggesting that instability is more likely than average response times are slower. The average price elasticity of participants is represented by the second most significant feature, *g_mean*, where more elasticity tends to support system stability. *tau_std*, which reflects variability in node response dynamics, is another important characteristic. These results show that system-level behavior beyond individual node attributes is successfully captured by the developed interaction features. Furthermore, the reliability of the evaluated models is validated by stratified 5-fold cross-validation. Stable generalization performance is indicated by ensemble and boosting approaches with low variance across folds. Because it reduces the risk of overfitting by aggregating predictions from a variety of base learners, the Hybrid Stacking Ensemble exhibits uniform behavior. Overall, the results show that while sequential deep learning architectures have limited benefits for tabular grid datasets, nonlinear ensemble approaches and hybrid learning frameworks offer the most dependable and comprehensible solutions for grid stability prediction. Table 6 provides a comparative table against existing works for predicting grid stability. The proposed approach exceeds the reported accuracy of other works. Beyond predictive accuracy, the proposed research work integrates SHAP-based interpretation using beeswarm, bar and dependence analyses, whereas the compared studies provide no XAI analysis or more limited interpretability.

**Table 5:** Performance comparison of machine learning and deep learning models for grid stability prediction.

Model	Accuracy	F1-score (W)	AUC-ROC	MCC	Prec. (stable)	Rec. (stable)	Prec. (Unstbl)	Rec. (Unstbl)	Train time (s)
Hybrid stacking ensemble	0.9685	0.97	0.99	0.93	0.95	0.96	0.98	0.97	75.70
Deep MLP	0.9680	0.96	0.99	0.93	0.95	0.96	0.98	0.97	22.80
SVM (RBF)	0.9635	0.96	0.99	0.92	0.95	0.95	0.97	0.97	5.10
XGBoost	0.9520	0.95	0.99	0.90	0.95	0.92	0.96	0.97	2.90
LightGBM	0.9515	0.95	0.99	0.89	0.94	0.93	0.96	0.97	2.20
Gradient boosting	0.9460	0.95	0.99	0.88	0.93	0.92	0.95	0.96	37.80
Random Forest	0.9230	0.92	0.98	0.83	0.91	0.88	0.93	0.95	15.10
1D-CNN	0.900	0.91	0.97	0.80	0.87	0.88	0.93	0.92	24.50
CNN-LSTM hybrid	0.8935	0.91	0.97	0.80	0.88	0.86	0.92	0.93	45.50
Logistic regression	0.8765	0.88	0.95	0.73	0.82	0.84	0.91	0.90	0.10
Bi-LSTM	0.8240	0.79	0.94	0.60	0.95	0.50	0.78	0.98	26.60

**Figure 2:**
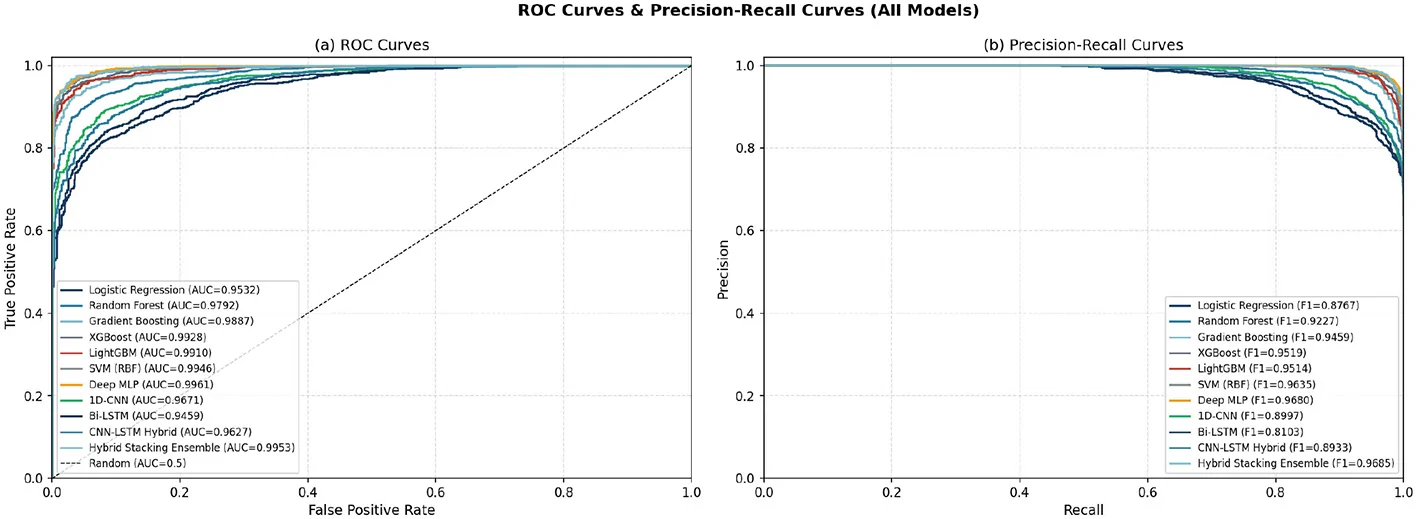
Receiver operating characteristic (ROC) **(a)** and Precision-Recall curves **(b)** comparing classification performance of the evaluated models.

**Figure 3:**
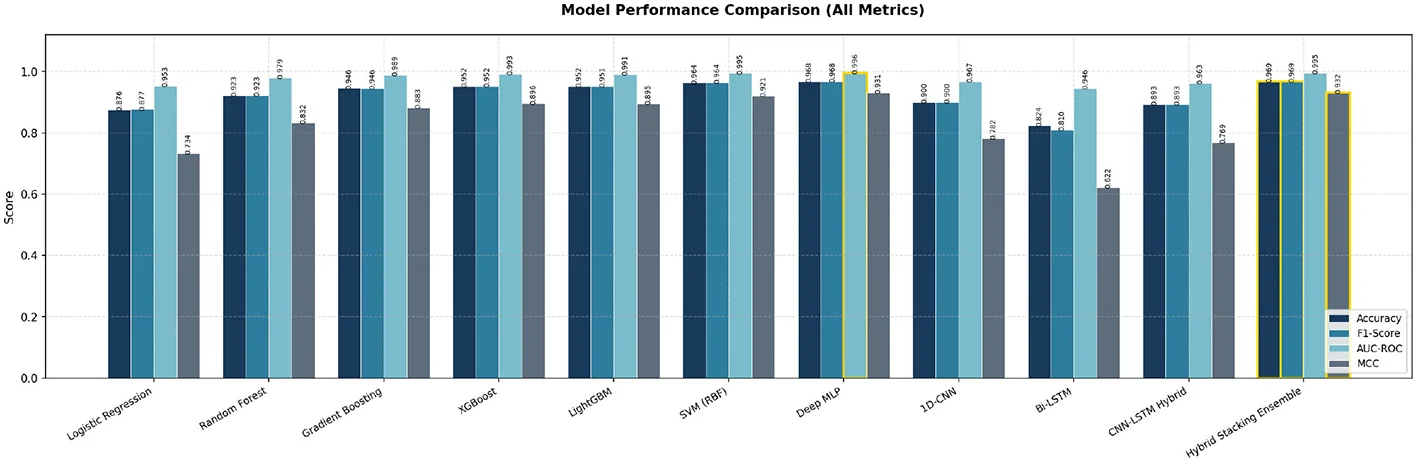
Comparison of model performance metrics including accuracy, F1-score, AUC–ROC, and MCC across different machine learning and deep learning models.

**Figure 4:**
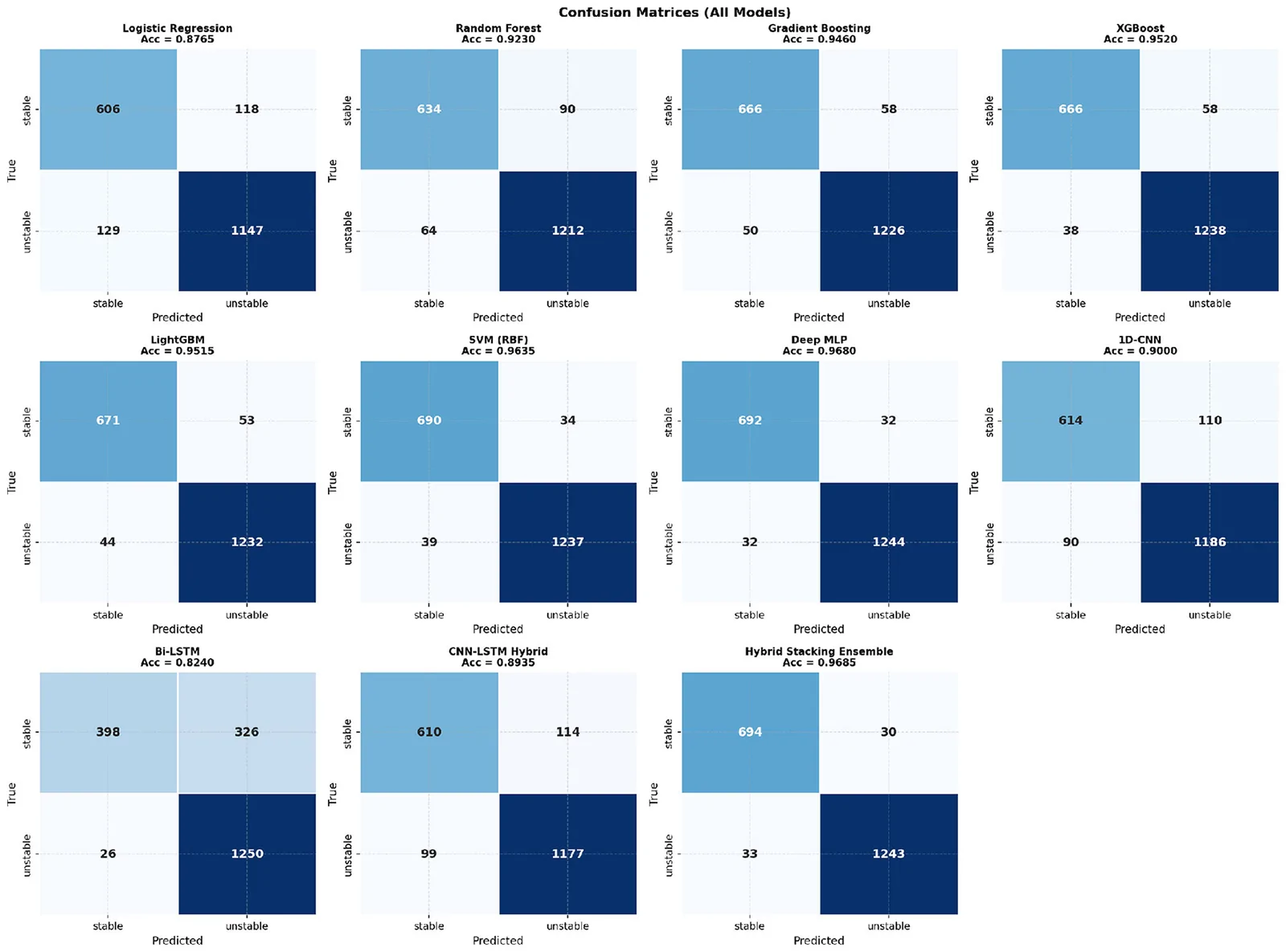
Confusion matrices of the evaluated models illustrating classification performance for stable and unstable grid states.

**Figure 5:**
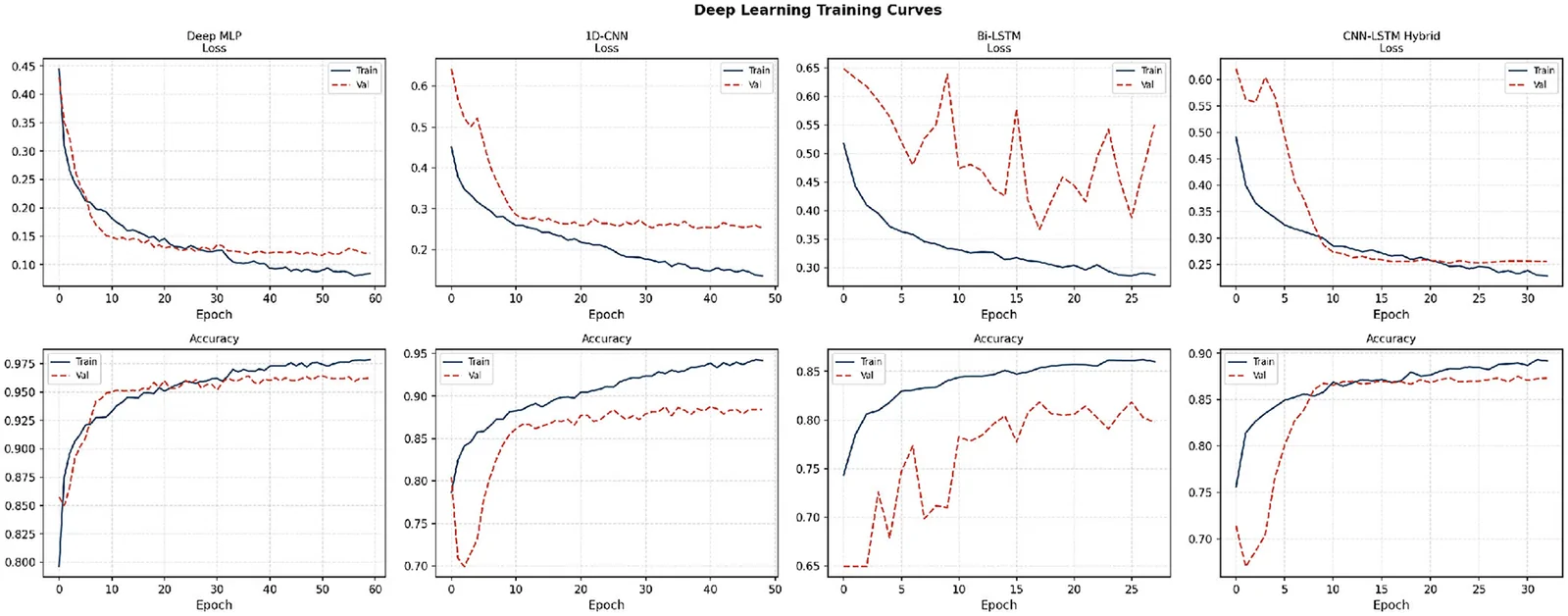
Training and validation curves illustrating the convergence behavior of the deep learning models during the training process.

**Figure 6:**
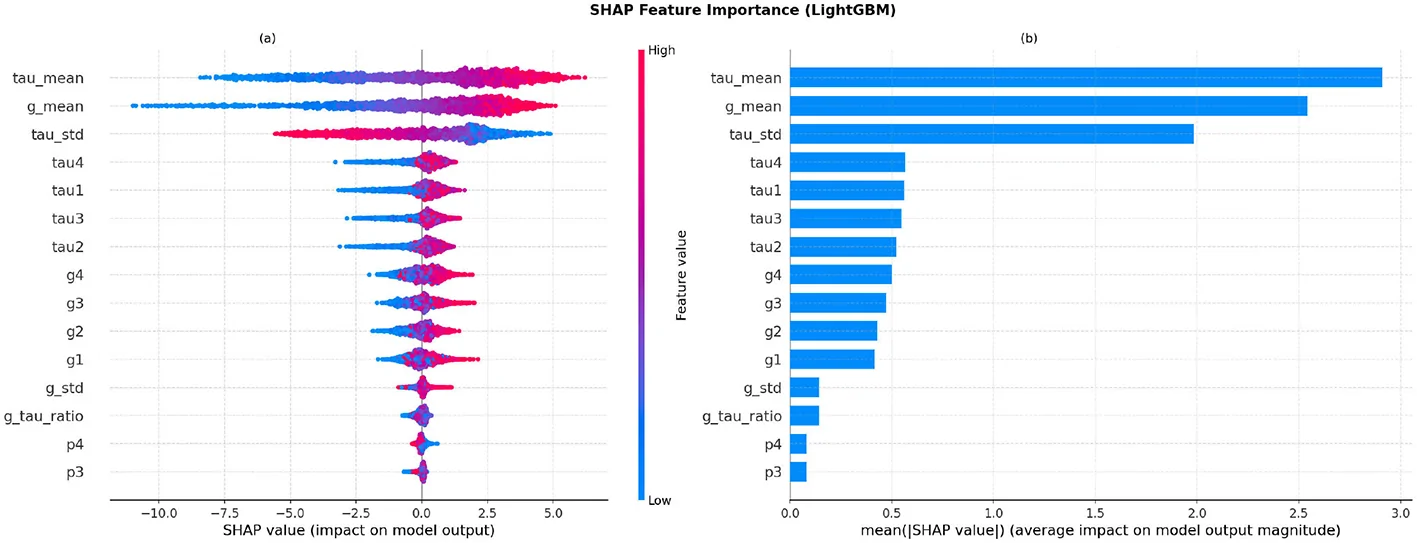
SHAP summary plot highlighting the most influential features affecting grid stability prediction. **(a)** SHAP Beeswarm—LightGBM. **(b)** Mean |SHAP| bar chart.

**Figure 7:**
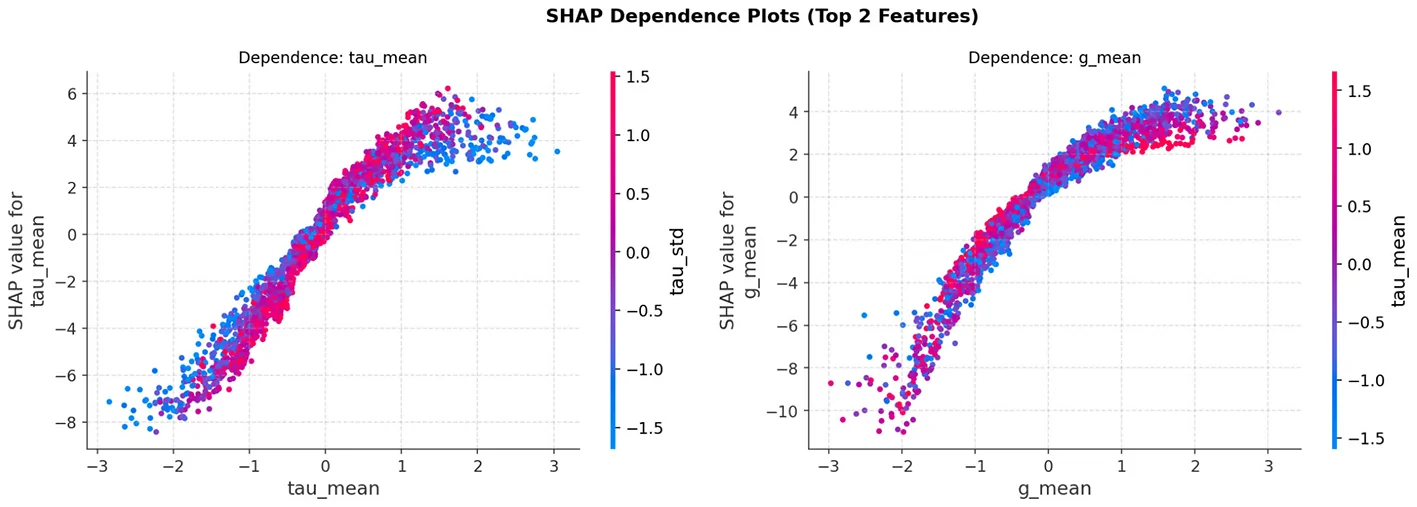
SHAP dependence plot illustrating the interaction between key engineered features and their impact on model predictions.

**Table 6:** Performance comparison of previous machine-learning models for grid stability prediction.

Study	Model	Accuracy (%)	XAI
Mostafa et al. (2022)	Penalized LR	96.00	None
Alsirhani et al. (2023)	MLP-ELM	95.80	None
Cifci (2025)	ANN	96.20	SHAP + ICE
Wang et al. (2025)	BO-SVM	96.00	SHAP
**Proposed**	**Hybrid stacking ensemble**	**96.85**	**SHAP (beeswarm + bar + dependence)**

SHAP, SHapley Additive exPlanations; SHAP-ICE, SHapley Additive exPlanations-Individual Conditional Expectation; Penalized LR, Penalized Logistic Regression; MLP, Multilayer Perceptron; ELM, Extreme Learning Machine; ANN, Artificial Neural Network; BO-SVM, Bayesian Optimization–Support Vector Machine.

A further limitation concerns the strength of the evidence for generalization. The present evaluation should not be interpreted as evidence of performance under different operating conditions or independently designed system configurations. More rigorous evidence would require repeated or nested cross-validation, independent re-simulation under unseen operating conditions, and ultimately, validation on alternative system configurations or real-world measurements. Accordingly, the reported performance should be interpreted as evidence of predictive ability within the evaluated dataset, rather than as a basis for claims about broader real-world deployment.

## 7 Conclusion

This study used a simulated decentralized response dataset to propose a machine learning structure for electrical grid stability prediction. Several performance indicators and interpretability studies were used to accurately evaluate a wide range of models, including Deep Neural Networks, Gradient Boosting approaches, conventional ML algorithms, and a Hybrid Stacking Ensemble. The results show that nonlinear learning techniques work better than linear models in capturing complex relationships and stability outcomes. With an accuracy rate near 96.85% and AUC-ROC scores of about 0.9953, the Hybrid Stacking Ensemble demonstrated the best prediction performance among the examined techniques. The efficiency of advanced ensemble models for structured grid data was demonstrated by the reliable and computationally efficient performance of kernel-based SVM and gradient boosting models like XGBoost and LightGBM.Stability prediction is dominated by engineered system-level properties, according to interpretability analysis utilizing SHAP. The most significant predictors were found to be the average reaction time (τ_mean_), elasticity parameters (*g*_mean_), and response time variability (τ_std_). These results show that collective network dynamics have a major impact on grid stability conditions, which is in line with theoretical assumptions in decentralized demand response systems. However, these attributions represent learned statistical associations rather than causal physical mechanisms. The results are interpreted with a 4-node dataset, although the raw stability margin was excluded to mitigate direct target leakage from engineered variables such as power balance and the τ-power interaction, which will remain correlated with the system parameters. Future studies will concentrate on expanding the structure to real-world power system datasets, including bigger multi-node energy systems and dynamic network topologies. The reliability and operational applicability of machine learning-based grid stability prediction will be further improved by combining temporal forecasting models with physics-informed learning techniques.

## Data Availability

The original contributions presented in the study are included in the article/supplementary material, further inquiries can be directed to the corresponding author.
